# Work-related risk factors and the prevalence of low back pain among low-income industrial workers in Bangladesh: results from a cross-sectional study

**DOI:** 10.1186/s43161-023-00132-z

**Published:** 2023-06-07

**Authors:** Md. Omar Sharif Ahmmed Chowdhury, Nurul Huda, Md. Mohsin Alam, Sayed Imran Hossain, Shohal Hossain, Shahana Islam, Most. Rumpa Khatun

**Affiliations:** 1 Medical Section, Administration Department, Walton Group, Gazipur, Dhaka, Bangladesh; 2 Bangladesh Cricket Board, Dhaka, Bangladesh; 3 Department of Communication Disorders, University of Dhaka, Dhaka, Bangladesh; 4 Bangladesh Naval Academy, Chittagong, Bangladesh; 5 Gono Bishwabidyalay, Savar, Dhaka, Bangladesh; 6 Physiotherapy Department, Rezia Taleb Hospital Ltd, Keraniganj, Dhaka, Bangladesh; 7 Physiotherapy Department, Gono Bishwabidyalay, Dhaka, Bangladesh

**Keywords:** Industrial worker, Low back pain, Work-related risk factors, Low wage workers, Prevalence, Bangladesh

## Abstract

**Objectives:**

Industrial workers are exposed to various musculoskeletal problems especially in tasks related to production. Low back pain is the main problem in most musculoskeletal disorders. Therefore, our study aims to identify the prevalence of work-related low back pain and its risk factors among industrial workers in Bangladesh.

**Materials and methods:**

A sample of 384 industrial workers aged 18–55 years in Bangladesh was selected to conduct this cross-sectional study to assess low back pain prevalence and identify its risk factors. Regression analysis was performed regarding socioeconomic status and risk factor related to LBP through interviews and questionnaires.

**Results:**

Data analysis showed that the prevalence of LBP in industrial workers in Dhaka City was 238 (62%). Low back pain was associated with increasing age (odd ratio = 1.05). Other significant risks for pain in the low back were being a permanent employee (OR 3.15) and working more than 8 h (OR = 1.99). Also, LBP was associated with incorrect body mechanics use, repetitive bending, and continuous long-time sitting risk was higher than others.

**Conclusion:**

The results of this investigation highlight a high proportion of LBP and its risk factors among industrial workers in Dhaka City. Age, type of employment, working hours, use of incorrect body mechanics, repeated bending or twisting, and prolonged sitting were among the risk factors for back pain. Therefore, exercising, providing adequate training for properly maintaining body mechanics, and avoiding bad positions may be among the most powerful steps needed to avoid or prevent development of back pain.

## Introduction

Low back pain (LBP) can be defined as chronic or acute pain that is felt in the lumbosacral, buttocks, or upper leg area of the human body, and lower back diseases are associated with work-related faulty postures [1]. The result of this issue is increased movement difficulty, disability, poor service, poor lifestyle, and absences for sickness in working places [2]. One of the greatest usually reported muscle or joint problem is low back pain, which is a the leading cause of burden on health systems, individuals, and the social care system [3]. Hazardous factors for pain in the lower back and its prevalence among workers are unknown in Bangladesh. One of the major public health problems is LBP, with over 80% of the world’s population reporting LBP at some point in their lives [4]. Especially in low- and middle-income countries, LBP prevalence is high [5]. Industrial workers in poor working conditions bear some responsibilities for LBP. Workers in any section of industries are often managing many unplanned works with various tasks and obligations [6]. Industrial work is a physically demanding job. Low back pain is related to a variety of occupations, such as driving, manual handling, operating, being a technician, general worker, cleaner, and many more occupations, that involve many inappropriate body movements, which in turn contribute to MSDs [7]. Work-related physical exposures include, especially standing or sitting for a long time, lifting heavily, cleaning tiles and vacuuming in every shift, repeatedly bending or leaning forward, managing manual instruments, working in faulty positions, and working out of physical capacity, working with full body vibration, to do Continuing to work despite injuries, lack of regular, adequate rest is a well-established risk factor for LBP in low-income industrial workers [8]. It forms easy to see that numerous injury types are straightly involved in the tasks performed in the industrial occupation [9]. The United States Housekeepers had the utmost total rate of injury and the maximum proportion of MSDs, most generally low back pain, among guest house workers [10]. LBP has been one of the leading causes of disease in developing countries than in underdeveloped countries in recent decades [11]. It is often seen that back pain and its frequency are considered trivial problems compared to other diseases that have a high mortality rate, like cancer or contagious diseases 12]. For the current lifestyle, low back pain has placed a significant economic burden on the government, especially in the areas of healthcare costs, reduced productivity, lost working days, and disability [13]. However, in the case of illness, the pain of back disorders is a leading effect of many factors, including the absence of work and the limitation of activities in the workplace. In addition to the extra human cost, back pain has a high impact on society’s resources [14].

Industrial work-related LBP is associated with disclosure to ergonomic pressure at work, physical, psychosocial, and/or individual hazard factors [15]. In some research, wide extents of effects related to low back pain have been characterized. Into these, carrying and lifting heavy contents [16]. The 2010 GBD (Global Burden of Disease) research approximates that LBP is among the major 10 disorders and injuries that are calculated for incapability-stable life years globally [17]. LBP affects not only the economic loss of workers and the quality of social life, such as the reduced capacity to work, decrease production, and early retirement but also the organization, society, and country as a whole [18]. Although there has been a lot of research on back pain in Bangladesh, among the several groups of workers in the bank, universities, and hospitals, such as the degree of back pain and related causes, no research has been done on industrial workers. Therefore, the research was driven to discover the prevalence and identify risk factors of low back pain among industrial workers in Dhaka, Bangladesh.

## Materials and methods

### Study design and participants

The study was selected as an organization-based cross-sectional study conducted from June 2022 to November, 2022. The research was driven at Gazipur area industries in Bangladesh. Gazipur is one of the leading industrial areas, which is the part of Dhaka division in Bangladesh. Data were collected from 384 participants aged 18 to 55 years industrial workers in Bangladesh who were apparently healthy and active in working. The Gazipur is the known industries area in Dhaka and workers are the largest population in this area.

### Source and study population

All workers who worked in Dhaka City were the source population. Workers in Gazipur area industries, who had worked at least 1 year in the industries, were included in this study. While, workers with spinal deformities (such as excessive kyphosis, lordosis, flat back, and scoliosis), inflammatory diseases, disc prolapse, or traumatic injury affecting the musculoskeletal system were excluded from the study.

### Determine sample size and procedures

The sample size was determined using a single population proportion formula, a 50% standard deviation, 5% margin of error, and 95% confidence interval resulting in 384 industrial workers randomly selected from 6 industries. A systematic randomize sampling technique was used to select the study respondent among the industries.

### Data measurements

A face-to-face interview was conducted while collecting data for this study and adequate precautions against COVID-19 were taken. The semi-structured questionnaire was developed primarily in English and later translated into Bengali. After the translation, the questionnaire was checked by another translator to check its accuracy. However, the local language was used to communicate with the respondents. Then, an orientation was organized among the collectors along with 38 (10%) field tests and finally, data was collected through door-to-door questionnaires from the industrial workers.

The project was organized in Sreepur, Chandra, Sofipur, Baroipara, Khamarbari, Kobirpur, Konabari, Kaliakoir, and Naojor areas of Gazipur City in Bangladesh. We collected data from them with their permission. The survey asked subjects, using only Bengali words, “Have you had pain or tenderness in your lower back most days or month (currently or in the past)?”. One response box had o on the questionnaire for the lower back of the body (yes and no).

### Statistical analysis

The *χ*2 test was used to see the relation of several factors with the magnitude of pain in the lower back. All answer sheets were checked for exactness, completeness, and internal consistency. Inconsistent data is discarded. Accurate data were entered into SPSS version 25 for analysis. We used logistic regression to evaluate the prevalence of specific low back pain in study populations and also fix to evaluate factors related to LBP. LBP was regressed against social-demographic, individual, and work-related factors differently. Prior fix the binary logistic regression, the quality of the model test was evaluated by Hosmer and Lemeshow test, and the hypothesis was satisfied (*p* value > 0.05). Qualitative data were used for thematic content analysis. Analyzes were limited to 384 Bangladeshi workers aged 18 to 55 years.

### Data quality control

During data collection, supervisors checked each completed questionnaire for completeness and consistency. Throughout the data collection period, the data collectors at each site were supervised and had regular meetings with the principal investigator.

## Results

All 384 completed and valid questionnaires were returned and considered for the analysis, which gives a response rate of 100%.

### Socio-demographic characteristics of the study respondents

The majority 331 (86.2%) of the study participants were males. A maximum of 307 (79.9%) of the respondents had more than 1 year of work experience in an industry job. The mean (± SD) age of the participants was 31.32 ± 6.98 years and above half 210 (54.7%) of participants had an age limit of 28 to 37 years. Over two-thirds of the respondents totaling 266 (69.3%) had an average monthly income below 12,999 BDT. Only 193(50.3%) workers have health and safety tanning, and most of the 325 (84.6%) respondent’s employment patterns are permanent (Table 1).

**Table 1:** Socio-demographic characteristics of the study respondents, Dhaka, Bangladesh (*n* = 384)

Characteristics	Overall	Male	Female
	*N* (%)	*N* (%)	*N* (%)
Sex			
	384(100%)	331 (86.2%)	53(13.8%)
Age group (in years)			
18–27	118(30.7%)	92 (24%)	26(6.8%)
28–37	210(54.7%)	188(49%)	22(5.7%)
38–47	47(12.2%)	43(11.2%)	4(1%)
48–59	9(2.3%)	8(2.1%)	1(0.3%)
Mean age in years (mean ± SD)	**31.32 ± 6.98**	**31.64 ± 6.94**	**29.28 ± 6.97**
Marital status			
Single	114(29.7%)	93(24.2%)	21(5.5%)
Married	270(70.3%)	238(62.0%)	32(8.3%)
Average monthly income			
8000–12,999	266(69.3%)	231(60.2%)	35(9.1%)
13,000–15,999	56(14.6%)	48(12.5%)	8(2.1%)
16,000–19,999	40(10.4%)	33(8.6%)	7(1.8%)
20,000 +	22(5.7%)	19(4.9%)	3(0.8%)
Employment pattern			
Temporary	59(15.4%)	37(9.6%)	22(5.7%)
Permanent	325(84.6%)	294(76.6%)	31(8.1%)
Specific work experience			
< 1 year	77(20.1%)	62(16.1%)	15(3.9%)
> 1 year	307(79.9%)	269(70.1%)	38(9.9%)
Working hours (per day)			
< 8 h	313(81.50%)	274 (71.4%)	39(10.20%)
> 8 h	71(18.5%)	57(14.80%)	14(3.60%)
Training on health and safety			
No	191(49.7%)	156(40.6%)	35(9.1%)
Yes	193(50.3%)	175(45.6%)	18(4.7%)

### Working status of the participants

Workers work in the workplace with various bad postures, which are revealed based on their work patterns and their interviews. About 52 (13.54%) of the industrial workers worked in a faulty position (like hanging and leaning forward) and 53 (13.80%) worked continuously in one position for long hours. Some workers were not pleased with their working environment. Among those who had pain proneness, 59 (15.36%) worked in forward bending or lying down and 57 (14.84%) sat for long periods of time (Table 2).

**Table 2:** Working position related characteristics of study from survey where survey data (*n* = 384)

Characteristics		Yes *N* (%)	No *N* (%)
Working in an faulty position	Male	41(10.7%)	290(75.5%)
	Female	11(2.9%)	42(10.9%)
	Overall	52(13.5%)	332(86.5%)
Stationary position for a long time	Male	48(12.5%)	283(73.7%)
	Female	5(1.6%)	48(12.5%)
	Overall	53(13.8%)	331(86.2%)
Incorrectly using body mechanics	Male	39(10.2%)	292(76.0%)
	Female	6(1.6%)	47(12.2%)
	Overall	45(11.7%)	339(88.3%)
Repeated bending and twisting	Male	52(13.5%)	279(72.7%)
	Female	7(1.8%)	46(12%)
	Overall	59(15.4%)	325(84.6%)
Working beyond physical ability	Male	24(6.3%)	307(79.9%)
	Female	5(1.3%)	48(12.5%)
	Overall	29(7.6%)	355(92.4%)
Continuously long time sitting	Male	49(12.8%)	282(73.4%)
	Female	8(2.1%)	45(11.7%)
	Overall	57(14.8%)	327(85.2%)
Continue work despite injury or pain	Male	17(4.4%)	314(81.8%)
	Female	2(0.5%)	51(13.3%)
	Overall	19(4.9%)	365(95.1%)
Sing ergonomically improper tools	Male	28(7.3%)	303(78.9%)
	Female	3(0.8%)	50(13%)
	Overall	31(8.1%)	353(91.9%)

### Prevalence of low back pain

**Fig. 1:**
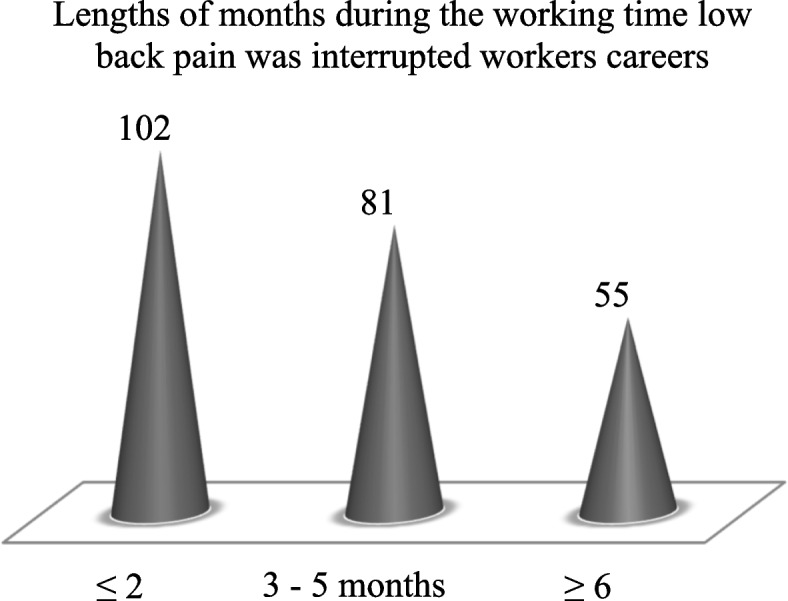
Lengths of months interrupted due to low back pain during workers job life

This research display that the prevalence of pain in the lower back among industry worker in the Dhaka area was 238 (62%). In addition, 238 (62%) of the workers had back pain affecting their ability to do normal work even if it lasted for at least 1 month during their working life. Of these, the majority of study workers 102 (42.86%) reported that low back pain had prevented them from working for at least 1 month or less during their job life (Fig. 1).

### Factors associated with low back pain

Logistic regression showed that age, type of employment, working hours, incorrect use of body mechanics, repeated bending or twisting, and prolonged sitting had a statistically significant association with low back pain (*p* value ≤ 0.05). However, no significant association was observed between gender, marital status, work experience, and training in health and safety with low back pain (Table 3).

**Table 3:** Logistic regression of socio-demographic characteristics and factors related characteristics

	OR (95% CI)	*p* value
Age in year	1.05 (1.01, 1.09)^*^	.017
Sex		
Male	Reference	
Female	1.94 (.97, 3.88)	.063
Marital status		
Single	Reference	
Married	1.37 (.76, 2.45)	.293
Employment		
Temporary	Reference	
Permanent	3.15 (1.55, 6.39)^***^	.002
Experience		
< 1 year	Reference	
> 1 year	1.07(.53, 2.13)	.856
Working hours		
< 8 h	Reference	
> 8 h	1.99(1.04,3.82)^**^	.039
Training on health and safety		
No	Reference	
Yes	1.42(.88, 2.31)	.154
Incorrectly using body mechanics		
No	Reference	
Yes	.027(.003, .209)^*^	.001
Repeated bending or twisting		
No	Reference	
Yes	.079(.021, .290)^*^	.000
Continuously long time sitting		
No	Reference	
Yes	.139(.027, .718)^*^	.019

This investigation showed that permanent employees had a higher risk of growing and becoming more mature back pain. Industrial job holders who have permanent job status had 3.15 times greater odds of having LBP when compared to temporary job holders (OR = 3.15, 95% CI 1.55, 6.39). Respondents working above than 8 h were 1.99 times more possible to have low back pain. OR = 1.99, 95% CI 1.04, 3.82). Younger workers were less possible to have back pain. Back pain was 1.05 times more likely with increasing age by 1 year OR = 1.05, 95% CI 1.01, 1.09).

According to this study, the workers worked in various postures in the industry. Their different body postures had been shown to be hazardous factors for LBP. Workers who were associated with using incorrect body mechanics, repeatedly bending or twisting the body, and sitting for long periods of time had a higher risk of back pain than others (OR = 0.027, 95% CI 0.003, 0.209), (OR = 0.079, 95% CI 0.021, 0.290)* and (OR = 0.139, 95% CI 0.027, 0.718)* were slightly higher. There is no relation between marital status, sex, and experience with increasing respondent’s having low back pain (OR = 1.94, 95% CI 0.97, 3.88), (OR = 1.37, 95% CI 0.76, 2.45) and for less than 1 year (OR = 1.22, 95% CI 0.64, 2.33), for more than 1 year (OR = 1.07, 95% CI 0.53, 2.13).

## Discussion

The objective of our investigation was to assess low back pain prevalence and its related factors. According to this survey, the prevalence of pain in the lower back was 238 (62%) among industrial workers. This investigation revealed that industrial workers are at higher risk for developing low back pain. This investigation has similarities with several studies. Among the studies, 60% of hotel workers in Malaysia [19], 46% of workers in the UK [20], 58.1% of workers in Ethiopia, and 60% of industrial workers in India [21] had low back pain prevalence. The results of this study support low back pain in workers. On the other hand, the results of this investigation are more commonly reported than studies conducted in other countries. However, a slightly higher prevalence than this study was reported in a study from Egypt, where the prevalence of pain in the lower back among workers was reported as 63.3% [22].

This study found that the prevalence of low back pain increased significantly with age. Industrial workers who were older had significantly higher rates of LBP than those who were younger. Our research data is consistent with reports from the Centers for Disease Control and Prevention. According to the CDC report, workers aged 45–64 were more likely to experience pain than younger workers [23].

This study found employment status in the industry to be an acceptable prophecy of back pain; Permanent workers were more likely to have back pain than temporary workers. A possible reason for this could be regular work for long periods of the year. Permanent workers have the same production workload throughout the year. Furthermore, groups of workers due to low income, work overtime to meet household expenses, promotions/increments, and long-term jobs [24]. Longer work hours were associated with low back pain. In this study, industrial workers who performed overtime duty or worked more than eight hours per day were significantly associated with rising low back pain. Due to the high workload in industries, many workers have to do extra work.

Low back pain was associated with worker workload. In this study, improper use of body mechanics is associated with rising pain in the lower back. Certain groups of industrial workers used incorrect body mechanics at work, making them more prone to low back pain than others. A possible reason for this may be that improper use of body mechanics requires loads on the back and muscular parts of the body, and pain increases with physical exertion. This study found that repetitive bending or twisting while performing tasks was a risk factor for low back pain. Hotel workers in Ethiopia who were associated with repetitive bending or twisting during work were more likely to develop back pain. This finding is similar to our findings. The low back pain risk was richer in employees who sat for long periods of time. A possible reason for this may be that sitting for a continuous prolonged time increases the tendency of pain in the lower back due to over-stretching or over-loading of the lower back muscles.

## Conclusions

In conclusion, the results of this study explain the prevalence of LBP and its risk factors among industrial workers in Dhaka City. This study showed a higher prevalence rate of LBP in industrial workers. Age, type of employment, working hours, incorrect use of body mechanics, repeated bending or twisting, and prolonged sitting were among the risk factors associated with low back pain. Therefore, taking breaks between tasks, exercising, providing adequate training for appropriate usage of body mechanics, avoiding repetitive bending or twisting, and prolonged sitting may be among the most powerful measures necessary to prevent low back pain.

## Data Availability

The data that take facilitate the discovery from this research will be made available from the corresponding author on logical reason. The data is not available publicly due to limitations in containing information that may lead to the privacy of the study participants and that may cause problems at the workplace.

## Abbreviations

LBP: Low back pain
MSDs: Musculo-skeletal diseases
OR: Odd ratio
GBD: Global Burden of Disease
SPSS: Statistical Package for the Social Sciences
SD: Standard deviation
BDT: Bangladesh Taka
CI: Confidence interval
UK: United Kingdom
CDC: Centers for Disease Control and Prevention
